# Considerations of strategies to provide influenza vaccine year round

**DOI:** 10.1016/j.vaccine.2015.08.037

**Published:** 2015-08-28

**Authors:** Philipp Lambach, Alba Maria Ropero Alvarez, Siddhivinayak Hirve, Justin R. Ortiz, Joachim Hombach, Marcel Verweij, Jan Hendriks, Laszlo Palkonyay, Michael Pfleiderer

**Affiliations:** a Initiative for Vaccine Research, World Health Organization, Geneva, Switzerland; b Immunization Unit, Pan American Health Organization, 525 Twenty Third St., NW, Washington, DC 20037, USA; c Global Influenza Programme, World Health Organization, Geneva, Switzerland; d Department of Social Sciences, Subdepartment Communication, Philosophy, and Technology, Wageningen University, Wageningen, The Netherlands; e Essential Medicines Department, World Health Organization, Geneva, Switzerland; f Federal Institute for Vaccines and Biomedicines, Paul-Ehrlich-Institut, Langen, Germany

**Keywords:** Year round, Supply, Influenza, Maternal, Tropics, Delivery, Logistics, Formulation

## Abstract

There is potential for influenza vaccine programmes to make a substantial impact on severe disease in low-resource settings, however questions around vaccine composition and programmatic issues will require special attention. Some countries may benefit from immunization programmes that provide year-round supply of vaccine; however the best way to ensure adequate vaccine supply has yet to be determined. In this report, we discuss vaccine composition, availability, and programmatic issues that must be considered when developing year-round influenza immunization programmes. We then explore how these considerations have influenced immunization practices in the Latin American region as a case study. We identify three different approaches to achieve year-round supply: (1) alternating between Northern Hemisphere and Southern Hemisphere formulations, (2) extending the expiration date to permit extended use of a single hemisphere formulation, and (3) local vaccine manufacture with production timelines that align with local epidemiology. Each approach has its challenges and opportunities. The growing data suggesting high influenza disease burden in low resource countries underscores the compelling public health need to determine the best strategies for vaccine delivery.

## 1. Influenza immunization in low- and middle-income countries

**Fig. 1.**
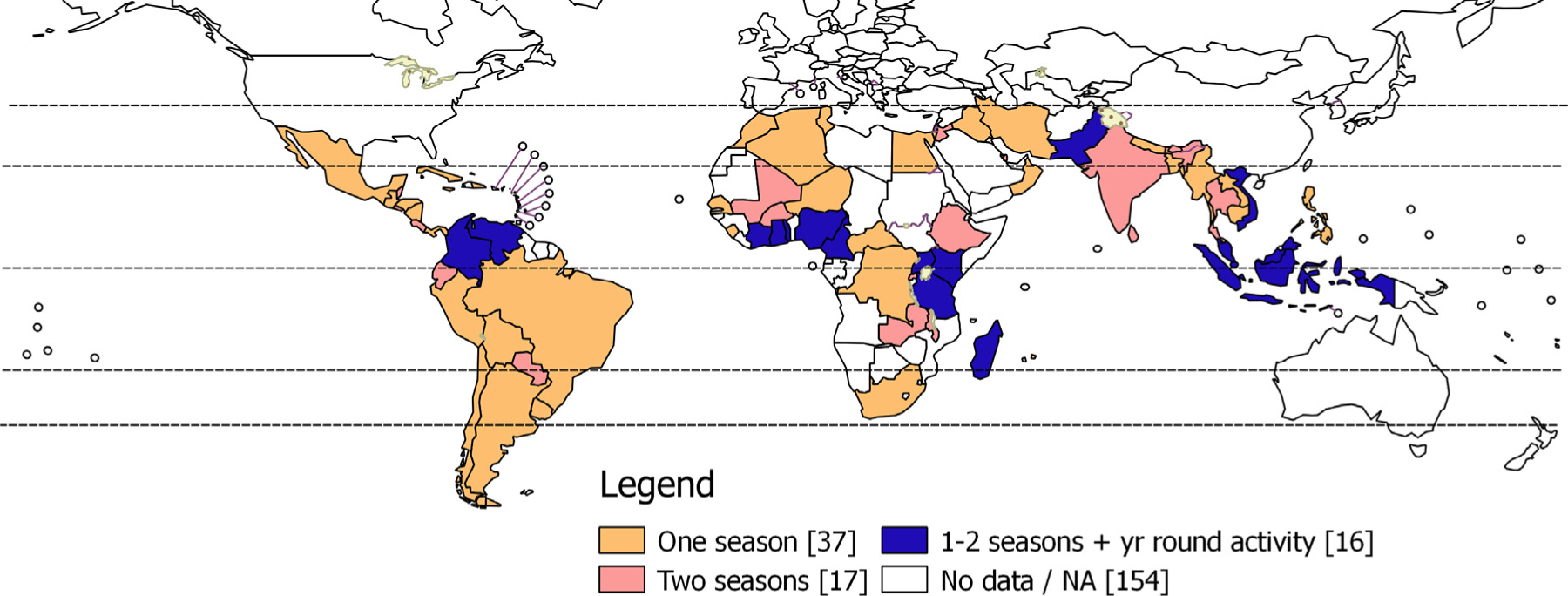
Influenza activity in countries across the world.

Seasonal influenza virus infection is associated with substantial morbidity and mortality worldwide [1–6]. Increasingly, low and middle income countries (LMICs) are recognized as having a high burden of severe influenza disease [1–6]. Many LMICs have influenza disease activity that is prolonged or that occurs during periods that differ from temperate country patterns (Fig. 1). There is potential for influenza vaccine programmes to make a substantial impact on severe disease in LMICs, however issues of vaccine composition, timing, delivery, and other programmatic issues in these settings will require special attention.

The World Health Organization (WHO) has identified young children, pregnant women, persons with chronic medical conditions, and the elderly as being at risk for severe influenza disease and therefore important groups to be considered for influenza vaccination [7]. Beginning in 2012, WHO recommended that pregnant women be prioritized for influenza vaccination by countries initiating or expanding influenza vaccine programmes. Many countries target some or all of these risk groups with annual mass vaccination campaigns that precede the anticipated beginning of the country’s influenza season. The ideal timing of influenza immunization in countries with prolonged or year-round influenza activity has yet to be determined, however year-round immunization programmes have been suggested as an approach to maximize disease prevention in these circumstances [8]. Even for countries with distinct, finite influenza seasons, there is a theoretical benefit to immunizing pregnant women year-round to provide protection to either the woman and/or the newborn—as one or both is bound to be exposed to the influenza season. While we are unaware of any countries that provide year-round influenza immunization, there is a compelling argument to assess the feasibility of this approach.

## 2. Key considerations for year-round supply of influenza vaccines

Many aspects of influenza disease and prevention have to be considered by countries when making decisions about immunization programmes, including disease burden, vaccine-specific issues, vaccine performance, vaccine safety, programme impact, programmatic issues, and country capacities and political will. In this report, we will focus on considerations of vaccine composition, availability, and programmatic issues. We will then explore how these considerations have influenced influenza immunization practices in the Latin American, the WHO region that most extensively uses influenza vaccine use.

### 2.1. Vaccine composition

Twice yearly, the WHO Global Influenza Programme leads a consultation of experts to recommend the composition of influenza vaccines based on the antigenic characteristics of circulating influenza viruses tested within the WHO Global Influenza Surveillance and Response System (GISRS) [9]. The Northern Hemisphere influenza vaccine composition is recommended in February and the Southern Hemisphere influenza vaccine composition is recommended in September. While most influenza isolates collected globally come from temperate regions, an increasing number of tropical countries are engaging in influenza surveillance and contributing to the GISRS process. Ensuring antigenic match of vaccine strains to circulating viruses is important to optimize vaccine performance. However, match does not necessarily correlate with vaccine effectiveness [10], and mismatched vaccines can still provide clinical protection [11]. In regions with influenza epidemiology that is not aligned with vaccine production cycles, the use of a vaccine with an extended shelf life may be therefore a viable strategy to protect vulnerable populations where no access to the most recent formulations exists.

### 2.2. Vaccine availability

**Fig. 2.**
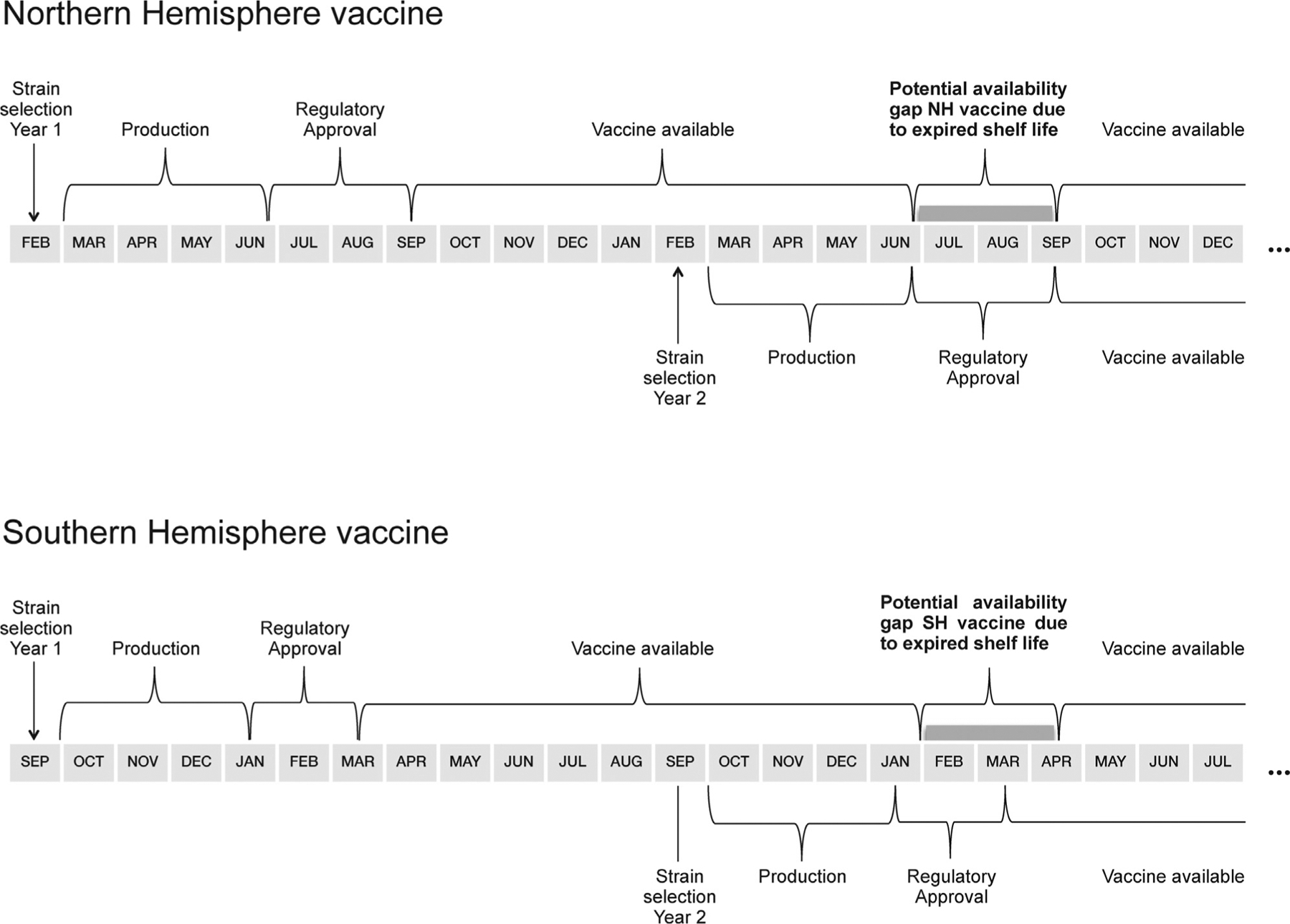
Schematic of seasonal influenza vaccine timelines.

Current influenza vaccine production cycles are based on the assumption of discrete and predictable annual influenza season peaks in temperate regions, as manufacturers have generally served those markets. Depending on regulatory and distribution processes, the Northern Hemisphere vaccine usually becomes available between August and September of a given year, while Southern Hemisphere vaccine usually becomes available March of the subsequent year [12,13] (Fig. 2). Since shelf lives of vaccine lots are defined by release dates following production and filling which are restricted to one year, the Northern Hemisphere vaccines typically expire by June or earlier, and the Southern Hemisphere vaccines typically expire by January or earlier. These expiration dates lead to ‘gap’ months occurring between the expiration date and the distribution of the next formulation of vaccine during which time no vaccine is available.

### 2.3. Programmatic issues

The WHO Extended Programme on Immunization (EPI) ensures that all low-resource countries have a strong platform for the administration of vaccines in children less than one year of age. As influenza vaccine is not licensed for use by children <6 months, and as influenza vaccine performance is suboptimal in young children, the EPI platform is traditionally not leveraged for influenza vaccine delivery [14–16]. Other high risk groups such as children less than one year of age, persons with chronic disease, and the elderly often do not have delivery platforms amenable to routine influenza immunization in many low-resource settings. Notably, some low-resource countries have been able to successfully expand EPI platforms to reach other risk groups, particularly in the Latin American region.

The WHO policy recommendation that pregnant women be prioritized for influenza vaccine receipt is based, in part, on the relative ease to reach this target population, e.g. through integration into antenatal care systems [17]. Nevertheless, much work is needed to determine the best way to reach pregnant women for immunization without adversely affecting routine care delivery and health systems.

There are numerous logistical and system challenges that must be overcome to achieve year-round influenza immunization. Procedures or capacities to ensure procurement, delivery, stock rotation, and regulatory processes to license both Northern and Southern Hemisphere vaccines on a regular and continuous basis have not been implemented many low resource countries. These challenges may represent substantial impediments to year-round delivery of the most recent vaccine formulation. Even wealthy countries in temperate regions typically do not have access to the most recent vaccine formulation from a different Hemisphere to provide to travellers or to respond to summertime outbreaks [18,19]. For these reasons, practical programmatic considerations must be taken into account with any influenza vaccine programme with year-round delivery or delivery outside the usual temperate country campaign periods.

Decision making about the best approach to maternal influenza vaccination involves potentially competing ethical values. Vaccine delivery early in pregnancy will provide greater benefit to the woman, as she will be protected longer during the duration of her pregnancy supporting early immunization if the primary goal is to maximize protection of the mother. On the other hand, available data indicate that third trimester immunization is associated with higher protective antibody titres in the newborn, supporting late immunization if the primary goal is to maximize protection in the newborn [20–22]. Immunization programmes may consider using antenatal care services, i.e. maternal and child health and immunization clinics that are well established in most low- and middle-income countries, or even child visits to target pregnant women. Globally, 82 percent of pregnant women have at least one antenatal care visit with a skilled healthcare provider [23]. However, only 54 per cent of pregnant women benefit from at least four antenatal visits [23]. If influenza vaccine were to be delivered through antenatal care services, missed opportunities for immunization, such as due to the unavailability of vaccine, should to be avoided [16]. In a low resource setting, it may be preferable to recommend vaccination at the first (and possibly only) antenatal care visit during pregnancy, instead of aiming for higher levels of protection if vaccination were postponed to use the most recent formulation. Further, to ensure protection of women at any stage of pregnancy, particularly in settings with limited access to antenatal care, influenza vaccine should therefore not only be made available before the peak of the influenza season, but whenever pregnant women present for antenatal care. There is likely to be trade-offs between operational feasibility (providing vaccine at the first opportunity during a pregnancy) and timing delivery to maximum protection of the mother or newborn.

## 3. Influenza vaccine delivery

### 3.1. Influenza epidemiology in the tropics

More than 40% of the world’s population lives in the tropical and subtropical countries facing a similar if not higher burden of influenza compared to temperate countries [25–27]. The timing of the biannual vaccine composition selection—production cycle has worked well for the distinct influenza seasons seen in the colder months of the temperate regions of the northern and southern hemisphere but poses challenges for the tropics. Multiple peaks are often seen in the tropics that frequently coincide with rainy seasons and secondary peaks during colder months [28]. Furthermore, countries nearer the equator (e.g. Kenya, Malaysia etc.) often have identifiable influenza activity year-round [4,29,30]. Countries that span large distance in latitude (Brazil, China, and India) pose a further challenge for vaccination timing and formulation choice due to within-country variation in the influenza seasonality pattern [31,32].

To respond effectively to influenza epidemics, countries with varying seasonality or year-round circulation of influenza, or countries with large latitudinal spread, may require specific procedures using alternate vaccination supply and timing. To decide on the optimal delivery strategy for their country, policy makers need to be aware of local seasonality and strain patterns to time the use of the most appropriate vaccine formulation as recommended by WHO. [33].

### 3.2. Ensuring vaccine match to circulating viruses

**Fig. 3.**
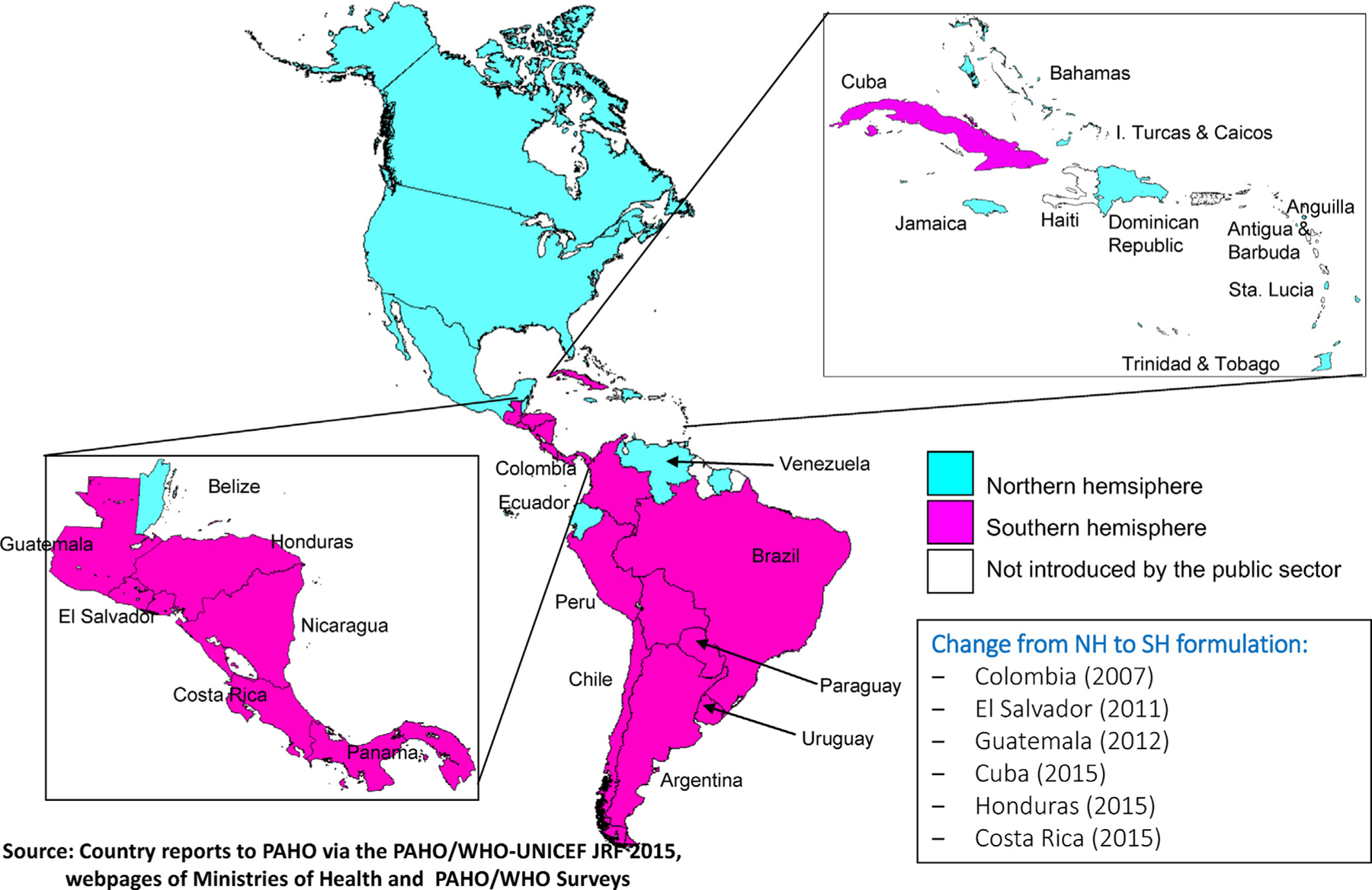
Use of seasonal influenza vaccine and formulation in the Americas.

Recent strengthening of influenza surveillance systems in Central American countries (Costa Rica, El Salvador, Guatemala, Honduras, Nicaragua, and Panama) have recently allowed for evaluation of optimal timing of influenza vaccines and formulation choice (Table 1). Surprisingly, availability of Southern hemisphere vaccine formulation has been found to align best with influenza disease epidemiology in many Central American countries compared to the Northern hemisphere vaccine formulation (the predominant strain was included in the vaccine in 81% as compared to 56% of the years surveyed based on epidemiologic data available from countries, as well as other analyses in the region). In response to these data, six tropical Latin American countries (Colombia, Costa-Rica, Cuba, El Salvador, Guatemala, and Honduras) modified their immunization policies, switching from Northern to Southern Hemisphere formulations between 2007 and 2015 [34] (Fig. 3).

### 3.3. Supply and delivery considerations

North and South America have the most experience targeting pregnant women for influenza vaccine receipt. Seasonal influenza immunization policies exist in 40 out of 45 countries and territories in the Americas. Of these, 27 identify pregnant women as priority groups for influenza immunization (Table 1). In Central America and South America, an ongoing study using laboratory-confirmed influenza like illness and severe acute respiratory-tract infection surveillance data from 2002 to 2013 (excluding 2009–2010) indicates that most countries have a distinct peak that is similar to the southern hemisphere seasonality pattern, with the exception of Belize, Costa Rica, Ecuador, El Salvador and Paraguay which have two peaks. Furthermore, Colombia and the Bolivarian Republic of Venezuela show year-round influenza circulation.

Although active licenses (i.e. marketing authorization) for seasonal influenza vaccines las up to a year for both the Northern or the Southern Hemisphere, there is a risk that no usable vaccine is available in regions falling outside the typical temperate country pattern during the ‘gap’ months. Policy makers in tropical countries wishing to protect populations from influenza have faced the challenge that circulation of influenza strains in their county may not coincide with peak seasons observed in temperate regions. This may potentially call for a different timing of vaccine campaigns, which should take into account ‘gap’ months during which neither the Northern or Southern vaccine formulation is available [33].

### 3.4. Global guidance on vaccine delivery

The WHO Immunization Practices Advisory Committee (IPAC) provides advice to WHO on programmatic aspects of vaccine delivery. Further advice of the IPAC and WHO is on the review and/or development of immunization practices, operational standards, tools and technologies necessary should inform national policy decisions. WHO guidance for the delivery of influenza vaccines year-round will be reviewed by IPAC for operational feasibility, programme quality, and access.

## 4. Strategies to ensure year-round supply of influenza vaccines

From a manufacturing and regulatory perspective, countries could choose between three strategies to ensure a year-round supply of vaccine depending on their capacity. While none of these approaches is currently being pursued, each has its merits and, when fully implemented, would ensure vaccine supply when vaccine is needed most.

### 4.1. Alternating between the most recent formulations when each is available

To ensure use of the latest vaccine formulation as recommended by WHO, a country can achieve year-round seasonal influenza vaccine delivery by alternating between Northern and Southern Hemisphere formulation throughout the year as each becomes available. This approach ensures immunization of the target population with the latest vaccine formulation available. This approach requires that national health systems have the logistical capacities to manage withdrawing a vaccine formulation in use as soon as the updated formulation becomes available and to deliver the latest formulation to their immunization programmes.

### 4.2. Extending the shelf life of vaccines to make the vaccine usable throughout the year

Extending the shelf life of influenza vaccines is an alternative approach to ensure vaccine supply year-round. Several considerations support this approach. First, even if the circulating influenza virus has drifted antigenically from the vaccine strain during the ‘gap’ months, the vaccine will still be able to provide protection against disease due to cross-protection with the drifted virus [11]. Second, seasonal influenza vaccines include strains against three or four different virus types and subtypes. Even in the event of antigenic drift of a circulating influenza strain, the vaccine can still be effective against the other circulating virus types/subtypes. For these reasons, health systems with limited capacities to manage logistical complexities of alternating influenza vaccines within the same year may consider extending the shelf life of vaccines as an alternative.

There are two ways that expiration dates are determined for influenza vaccines. In the United States, the expiration of influenza vaccines is set at June 30 of the year after production. In Europe, expiration dates are determined to occur one year after final vaccine vial filling. Both approaches arbitrarily choose expiration dates to facilitate stock rotations during periods of low influenza activity in anticipation of the availability of the next vaccine formulation. Manufacturing countries in temperate regions mainly limit expiration dates to below twelve months to ensure stock rotations, reducing the risk of stock overlap and the use of superseded vaccine formulations. In contrast, available data confirm the stability of influenza vaccines beyond 12 months post final vaccine vial filling [35]. To minimize possible overlap in use between previous and subsequent formulations, the extension of shelf life should be limited to 3 months.

From a manufacturing and regulatory perspective, an extension of the shelf life of vaccines can be achieved in two ways. In manufacturing countries where regulators prescribe twelve month post-production shelf-life duration, vaccine that is produced and released late in the production cycle could be designated for use in regions requiring later expiration dates. This approach would require minimal regulatory and logistic effort, as re-labelling would be avoided and filling lines would be less busy during the months of production. Alternatively, in countries where expiration dates are set at June 30, these dates could be extended by regulators of the manufacturing countries. This approach is feasible, although it would require relabeling of expiration dates for domestic and export markets, and regulators would likely require product-specific stability data beyond twelve months.

Through both approaches, influenza vaccines with shelf lives extended by additional three months to a shelf life of 15 months could ensure vaccine availability when it is most needed, e.g. for the Northern hemisphere during the potential ‘gap’ months between end of June and end of September.

### 4.3. Local production of vaccine

As a third supply option, local vaccine manufacturers could meet demands of tropical countries producing influenza vaccine to meet the needs of local disease epidemiology. In particular for low-income countries, this strategy may be a flexible, cost-effective and time-saving alternative. To support the decision making towards such a supply option, an in depth evaluation of economically sustainable local production and an assessment of the necessary regulatory requirements should be considered. Under the Global Action Plan for Influenza Vaccines (GAP) [36–38] the WHO is supporting the establishment and improvement of influenza vaccine production capacity in several low- and middle-income countries [38], mainly located in the tropics and subtropics. Year-round supply of influenza vaccines may contribute to significant public health gains. Several manufacturers in Asia and South America are now also producing seasonal influenza vaccines for domestic markets. For example, vaccine demands in Argentina, Brazil, and Mexico are served with locally produced vaccines [38–41].

## 5. Conclusion

This report describes considerations taken by WHO regarding promotion of year-round access to influenza vaccines. More data are needed to better understand the programmatic feasibility of alternating between Northern and Southern Hemisphere formulations, particularly in low resource settings without strong regulatory, procurement, delivery, and stock rotation. Further, more data regarding the relative differences in clinical protection afforded by rotating between hemisphere formulations versus using a single hemisphere formulation with an extended shelf life should be further evaluated. The growing data in favour of high disease burden in LMIC underscores the compelling public health need to determine the best targets for immunization and procedures to deliver vaccine.

## Figures and Tables

**Table 1:** Countries with influenza immunization policies in WHO/PAHO (total = 44 countries).

Number of countries with:	2004	2008	2013
Vaccination of healthy children	6 (14%)	22 (50%)	25 (57%)
Vaccination of children with chronic diseases only	–	–	5 (11%)
Vaccination of elderly	12 (27%)	33 (75%)	38 (86%)
Vaccination of persons with chronic diseases	9 (20%)	24 (55%)	35 (80%)
Vaccination of health workers	3 (7%)	32 (73%)	38 (86%)
Vaccination of pregnant women	3 (7%)	7 (16%)	27 (66%)

*Source:* Country reports to PAHO Joint Reporting Form (JRF), web pages of Ministries of Health, Pan American Health Organization/World Health Organization Surveys. Note: Data were not collected from the French Departments (French Guyana, Guadeloupe, Martinique).
